# Resistance mechanisms for Gram-negative bacteria-specific lipopeptides, turnercyclamycins, differ from that of colistin

**DOI:** 10.1128/spectrum.02306-23

**Published:** 2023-10-26

**Authors:** Albebson L. Lim, Bailey W. Miller, Zhenjian Lin, Mark A. Fisher, Louis R. Barrows, Margo G. Haygood, Eric W. Schmidt

**Affiliations:** 1 Department of Medicinal Chemistry, University of Utah, Salt Lake City, Utah, USA; 2 Department of Pathology and ARUP Laboratories, University of Utah, Salt Lake City, Utah, USA; 3 Department of Pharmacology and Toxicology, University of Utah, Salt Lake City, Utah, USA; University of Manitoba, Winnipeg, Manitoba, Canada

**Keywords:** lipopeptides, antibiotics, Gram-negative bacteria, *Acinetobacter*, colistin, drug resistance, turnercyclamycin

## Abstract

Turnercyclamycin A and B lipopeptides exhibit Gram-negative bacteria-specific toxicity. This includes CDC urgent threat organisms such as multidrug-resistant *Acinetobacter baumannii*. Like the last-line agent colistin, turnercyclamycins interact with the lipopolysaccharide (LPS) pathway, but they remain active against clinical isolates that are colistin resistant. Here, we aimed to determine why turnercyclamycins A and B show little cross-resistance with colistin despite some potential mechanistic and structural similarities. The outer membrane was important in the actions of all three agents, since the deletion of *lpxC* that synthesizes LPS and the addition of exogenous LPS led to resistance to turnercyclamycins and colistin. Even so, it was much more difficult to generate resistance to turnercyclamycin A than turnercyclamycin B. In *Escherichia coli*, disruption of the *mlaA* gene, which is involved in outer membrane homeostasis, resulted in resistance to turnercyclamycin B. However, *mlaA* disruption blocked neither turnercyclamycin A nor colistin activity. This activity was recapitulated in *A. baumannii*, where transposon mutants were more resistant to turnercyclamycin B. Moreover, the common *A. baumannii* colistin resistance gene *mcr-1* blocked colistin activity in *E. coli* but did not affect turnercyclamycins. These results demonstrate a unique resistance profile for turnercyclamycins A and B, in addition to suggesting differences in the mechanism of action. Further, turnercyclamycin A was effective in a mouse model of *A. baumannii* infection, indicating that this compound class may have potential promise in treating drug-resistant infections.

**IMPORTANCE:**

Bacterial resistance to antibiotics is a crisis. *Acinetobacter baumannii* is among the CDC urgent threat pathogens in part for this reason. Lipopeptides known as turnercyclamycins are produced by symbiotic bacteria that normally live in marine mollusks, where they may be involved in shaping their symbiotic niche. Turnercyclamycins killed Gram-negative pathogens including drug-resistant *Acinetobacter*, but how do the mechanisms of resistance compare to other lipopeptide drugs? Here, we define resistance from a truncation of MlaA, a protein involved in regulating bacterial membrane phospholipids. Intriguingly, this resistance mechanism only affected one turnercyclamycin variant, which differed only in two atoms in the lipid tail of the compounds. We could not obtain significant resistance to the second turnercyclamycin variant, which was also effective in an infection model. This study reveals an unexpected subtlety in resistance to lipopeptide antibiotics, which may be useful in the design and development of antibiotics to combat drug resistance.

## INTRODUCTION

Antimicrobial resistance is a global problem with Gram-negative pathogens dominating the list of urgent threats that needs to be addressed (1 – 4). For example, *Acinetobacter* is a particularly challenging pathogen that is intrinsically hard to combat due to the stability of its outer membrane and the ability to infect multiple sites (5 – 9). Multiple strains of drug-resistant *Acinetobacter* have emerged, and the underlying mobile resistance elements are easily transferred between strains (10 – 13). *Acinetobacter* is increasingly resistant to the last line of treatment, colistin (polymyxin E) (14 – 16). The polymyxin mechanism of action involves a portion of lipopolysaccharide (LPS), lipid A, in the outer membrane (OM) of Gram-negative bacteria. Polymyxins bind to lipid A through both lipid-lipid affinity and electrostatic interactions between polycationic polymyxins and the negatively charged phosphate groups of lipid A. The current model is that this interaction disrupts the outer membrane, displacing cations and lysing cells (17 – 20). As such, Gram-negative pathogens usually evade polymyxins by modifying negatively charged points of interaction (17, 18).

However, it is not clear that this simple mechanism fully explains the effectiveness of colistin and other lipopeptides. Recent studies have shown that lipopeptide antibiotics do not share a single mechanism but instead have different targets (17, 18). For example, colistin exhibited antibacterial activity by interacting with LPS in the inner membrane (IM), implying a different mechanism of action than direct interaction with OM LPS (21). Another study suggested that colistin forms free radicals, which would eventually induce oxidative damage to the cell (22). The lipopeptide brevicidine was recently found to interact with OM LPS but also to associate with phosphatidylglycerol and cardiolipin in the IM, resulting in bacterial stress leading to death (23). Other recent studies describe new lipopeptides with distinct mechanisms of action (24, 25). The unclear mechanism of colistin, the diversity of lipopeptide targets, and the prevalent resistance against colistin strengthen the need to discover lipopeptides with high therapeutic indices and distinct mechanisms of action.

**Fig 1:**
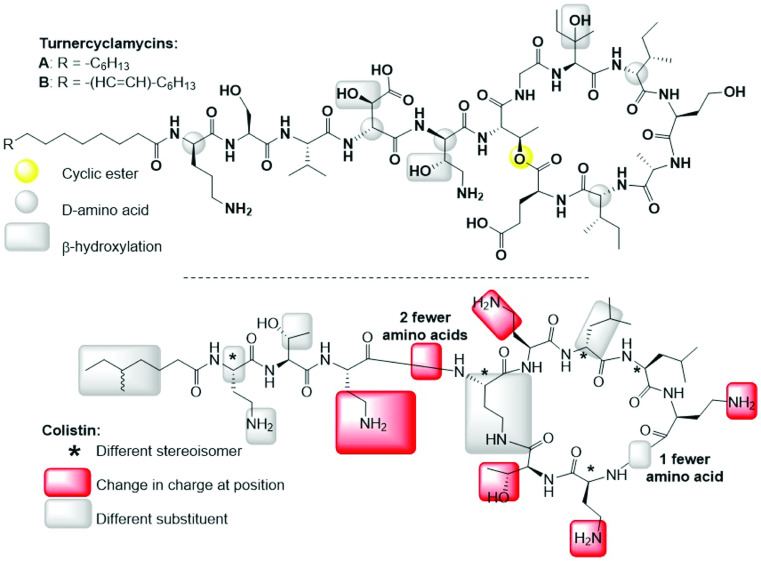
Structural comparison of turnercyclamycins and colistin (polymyxin E), the last-line agent for treating *Acinetobacter* infections. A key difference is that colistin is thought to act in part because it is polycationic, which might be important in binding to negatively charged lipids. In contrast, turnercyclamycins are net neutral in charge.

We have sought novel antibiotics by investigating natural biological interactions where antibiosis is likely to be important. One such model is shipworm mollusks, which harbor intracellular symbiotic bacteria rich in antibiotics (26 – 30). A common shipworm symbiont, *Teredinibacter turnerae*, is essential to the nutrition of the host mollusk, producing cellulases used for wood consumption (31). All *T. turnerae* strains examined so far also have the genetic capacity to produce lipopeptide antibiotics, the turnercyclamycins (Fig. 1) (30). While turnercyclamycins generally share identical peptide motifs, they differ in the length and unsaturation level of the lipid chain (30).

Turnercyclamycins showed promise in initial studies where they were active against Gram-negative pathogens without hemolysis or toxicity to kidney cells, which are major liabilities of lipopeptides (30). More importantly, both turnercyclamycins A and B were active against all tested clinical multidrug-resistant *Acinetobacter* strains (30). Despite their ability to kill colistin-resistant strains, initial studies revealed some effects that were very similar to those of colistin, potentially involving LPS and OM disruption, while other effects appeared to be unique to turnercyclamycins (30). Unlike polymyxins, where positive charge is thought to be important, turnercyclamycins are net neutral, implying either that the polycationic nature of polymyxins is unimportant or that turnercyclamycins may have different mechanisms in comparison to polymyxins. Thus, it was clear that despite some similarities, turnercyclamycins and colistin had differences in mechanisms of action and resistance.

Because antibiotic resistance is a crucial problem in therapy, here, we aimed to determine why turnercyclamycins and colistin did not show cross-resistance in clinical Gram-negative pathogens that we have tested previously. We show that the resistance mechanism is different not just for turnercyclamycins compared to colistin but that all three compounds have different resistance profiles and mechanisms. It was difficult to isolate mutants resistant to turnercyclamycin A, which is effective *in vivo*, suggesting a potential role for the turnercyclamycin scaffold in therapeutic development.

## RESULTS

### Turnercyclamycins are active against MCR-1-expressing bacteria

The plasmid-borne mobile colistin resistance gene (*mcr-1*) is a common mechanism of resistance against the polymyxin class of antibiotics (32, 33). The MCR-1 protein adds phosphoethanolamine to a phosphate group in lipid A. Because colistin is positively charged, this is thought to create an unfavorable charge interaction. In contrast, turnercyclamycins are net neutral (Fig. 1) (30). In our study, an *mcr-1*-expressing plasmid (Addgene pGDP2-MCR-1) was transformed into two strains of *Escherichia coli*: DH5α and C600. *E. coli* was used in place of *Acinetobacter* because both colistin and turnercyclamycins are very effective at killing *E. coli* (30), because *mcr-1* is reported to work by the same mechanism to provide *E. coli* with resistance against colistin (34), and because of the ease and relative safety of working with *E. coli*. The wild-type and *mcr-1* strains were treated with various concentrations of colistin, turnercyclamycin A, and turnercyclamycin B.

Colistin killed both *E. coli* parent and vector-control strains at low concentrations (MIC_90_ 0.5 µg/mL), but when tested against strains expressing *mcr-1*, it was 16-fold less potent (MIC_90_ 8 µg/mL) (Table 1). Both turnercyclamycins exhibited MIC_90_s of 2 µg/mL against the wild-type strains, which was not significantly changed in strains expressing *mcr-1*. From these results, we inferred that turnercyclamycins differ in mechanism compared to colistin, in terms of MCR-1-induced resistance. Additionally, these results paved the way for the possibility of turnercyclamycins being an alternate compound to combat colistin-resistant bacterial infections.

**TABLE 1:** Effect of the *mcr-1* resistance gene on colistin and turnercyclamycin susceptibility in *E. coli* DH5α and *E. coli* C600

Compounds	MIC_90_ (µg/mL)					
	DH5α	pBR322 in DH5α	pGDP2-MCR-1 in DH5α	C600	pBR322 in C600	pGDP2-MCR-1 in C600
Colistin	0.5	0.5	8	0.5	1	8
Turnercyclamycin A	2	2	2	2	4	4
Turnercyclamycin B	2	2	4	4	4	4

### LPS loss attenuates turnercyclamycin potency

**Fig 2:**
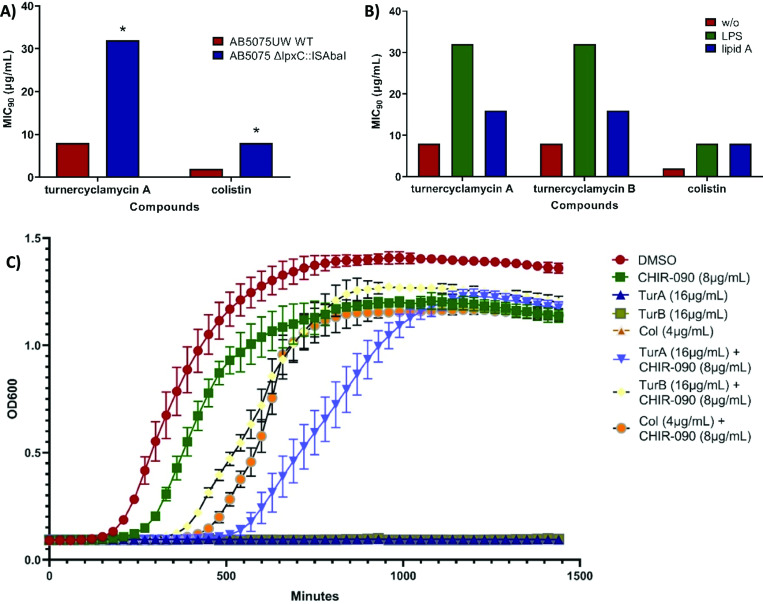
LPS is involved in turnercyclamycin activity. (**A**) MIC_90_s of turnercyclamycin A and colistin against wild-type (WT) and ΔlpxC *A. baumannii* 5075UW. * indicates that MIC_90_s are greater than or equal to the value given. (**B**) MIC_90_s of turnercyclamycins and colistin against *A. baumannii* after the addition of exogenous purified LPS and lipid A, both at 100 µg/mL. (**C**) Growth curve of *A. baumannii* measured using OD_600_, treated with the corresponding compounds. Each curve represents experiments performed using three biological replicates, with error bars indicating three replicate wells per biological replicate. The traces for turnercyclamycins A and B and colistin without other additives are difficult to see because they overlap on the baseline due to a lack of observable growth when treated with antibiotics. TurA and TurB, turnercyclamycins A and B; Col, colistin.

Initial studies suggested that LPS and lipid A might be potential targets of turnercyclamycins, since the susceptibility of *Yersinia pestis* to both colistin and turnercyclamycins was blocked by LPS modification with 4-amino-4-deoxy-L-arabinose (30). To test if LPS or lipid A plays a role in turnercyclamycin potency, as is the case with colistin, we determined the MIC of turnercyclamycin A against an *Acinetobacter baumannii* transposon mutant that is devoid of LpxC function (AB5075UW Δ*lpxC*::ISAbaI). LpxC performs the first committed step in lipid A biosynthesis, and therefore, its disruption produces a bacterium that is devoid of an LPS layer (35). Unlike many bacteria that require LPS/lipooligosaccharide (LOS) for survival, *Acinetobacter* remains viable without these lipids (36). When treated with turnercyclamycin A or colistin, *A. baumannii* Δ*lpxC* exhibited a greater than fourfold loss in susceptibility in comparison to its actions on the wild-type strain (Fig. 2A). This result is consistent with a role for LPS and lipid A in turnercyclamycin’s mechanism, similar to colistin.

To confirm this finding, we tested whether turnercyclamycins would kill wild-type *A. baumannii* following LpxC inhibition by a small molecule, CHIR-090 (37). As expected, CHIR-090 alone did not cause the death of *A. baumannii* since LPS is not required for survival, while turnercyclamycins and colistin were effective as single agents. However, when *A. baumannii* was treated with both CHIR-090 and either turnercyclamycins or colistin, the bacteria survived (Fig. 2B). Thus, both genetic knockout and chemical inhibition of LpxC yielded bacteria resistant to both families of lipopeptides.

Colistin loses efficacy in the presence of exogenously supplied LPS or lipid A, presumably because of competition with the OM-associated LPS for colistin binding (38, 39). Here, as expected, treatment of *A. baumannii* with 500 µg/mL of exogenous lipid A or LPS increased the MIC_90_ of colistin by approximately fourfold (Fig. 2C). In contrast, the MIC_90_s for turnercyclamycins A and B were increased by fourfold in the presence of intact LPS but only by twofold in the presence of purified lipid A. This result, coupled with the *mcr-1* resistance assay, led us to further investigate the resistance mechanisms through the generation of turnercyclamycin-resistant strains.

### Disruption of MlaA confers resistance to turnercyclamycin B but not A

**Fig 3:**
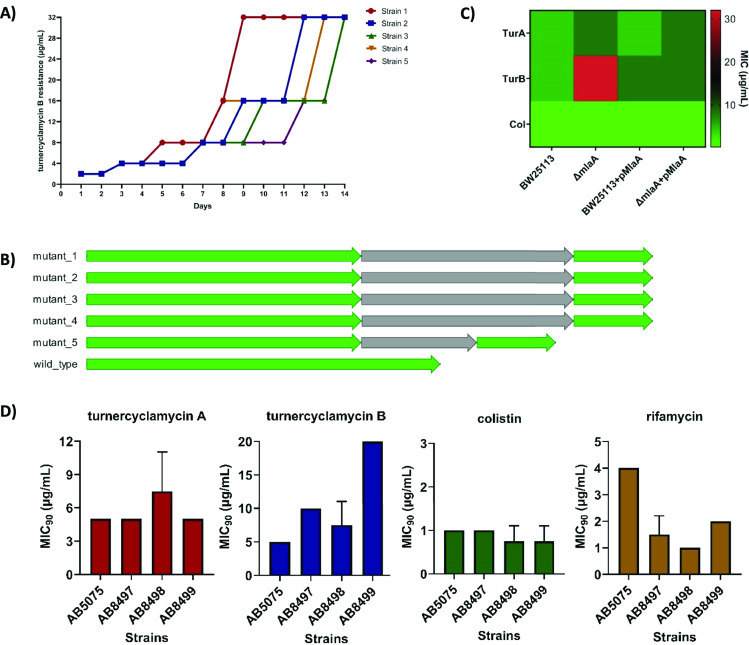
Disruption of MlaA confers resistance to turnercyclamycin B. (**A**) Turnercyclamycin B-resistant mutants were generated by daily serial passaging of *E. coli* DH5α at sub-MIC concentrations of turnercyclamycin, leading to a steady shift in MICs of turnercyclamycin B. This experiment was performed in five biologically independent samples. All mutants conferred early truncation mutations in MlaA. (**B**) Alignment of the *mlaA* gene in turnercyclamycin B-resistant mutant strains and WT, viewed using Geneious v10.2.2. All mutants showed early truncation caused by an insertion sequence (gray arrows) in the middle of the *mlaA* protein coding sequences (green arrows). (**C**) MICs of Keio *E. coli mlaA* knockouts treated with turnercyclamycins A and B and colistin, as well as rescue of antibiotic activity by introduction of the pMlaA complementation plasmid. All assays were done in triplicate and three biological replicates. (**D**) MICs of WT (AB5075) *A. baumannii* and *mlaA* knockout strains (AB8497–AB8499) treated with turnercyclamycins A and B, colistin, and rifamycin. All assays were done in triplicate and two biological replicates.

We used *E. coli* in our efforts to generate turnercyclamycin-resistant strains due to the large database of characterized genes and proteins when compared to *Acinetobacter*. Furthermore, turnercyclamycins are more potent against *E. coli,* making them preferred in assays requiring large amounts of compound. We aimed to perform serial inoculation of *E. coli* cultures into media with gradually increasing levels of turnercyclamycins per passage until an MIC_90_ of 32× the original MIC_90_ was achieved. *E. coli* was passaged daily for up to 63 d. While *E. coli* readily achieved this 32× target after 8–14 d of exposure to turnercyclamycin B (Fig. 3A), *E. coli* did not achieve the target level of resistance to turnercyclamycin A after 63 d (only 8× more resistant; Fig. S2). This suggests that turnercyclamycin A does not readily select for resistant mutants. Growth curves generated for the resistant strains revealed that they grow more slowly than the wild type (Fig. S1). Interestingly, even though turnercyclamycins A and B are extremely similar chemically, the turnercyclamycin B-resistant strains were actually more sensitive to turnercyclamycin A than was the wild type (Table 2; Fig. S3). Of note, despite the MIC_90_ of turnercyclamycin B being 32× that of the original, the MIC_50_ was still ~2 µg/mL (Fig. S3), indicating residual growth inhibition.

**TABLE 2:** MIC_90_s of turnercyclamycins A and B against evolved turnercyclamycin B-resistant *E. coli* DH5α

DH5α strain	MIC_90_ (µg/mL)		
	Turnercyclamycin A	Turnercyclamycin B	Colistin
WT	2	2	0.5
Strain 1	≤0.5	≥64	0.5
Strain 2	≥64	≥64	0.5
Strain 3	≤0.5	≥64	0.5
Strain 4	1.0	≥64	0.5
Strain 5	1.0	≥64	0.5

Illumina sequencing of turnercyclamycin B-resistant strains revealed that they all shared a mutation in a single gene encoding the MlaA protein (Fig. 3B). This mutation comprised an early termination of the *mlaA* gene caused by an insertion sequence, similar to hypothetical proteins found in *E. coli*, at site 589 (Fig. 3B). MlaA is an OM protein that maintains the asymmetry of the Gram-negative bacterial OM via retrograde trafficking of phospholipids from the OM to the IM (40, 41). To test whether disruption of MlaA was solely responsible for the loss of turnercyclamycin B activity, we tested both turnercyclamycin analogs and colistin against *mlaA* knockouts of *E. coli* from the Keio repository (42). Both turnercyclamycin A and colistin retained activity against the knockouts, with MIC_90_s of 8 and 0.25 µg/mL, respectively (Fig. 3C; Fig. S5). On the other hand, the turnercyclamycin B’s MIC_90_ was ≥32 µg/mL, with the same incomplete inhibition phenotype that was observed in the spontaneous mutants (Fig. 3C; Fig. S5).

We next transformed both wild-type (WT) and Δ*mlaA E. coli* with an MlaA expression plasmid and used the resulting strains in tests of antibiotic susceptibility. Expression of MlaA in the Δ*mlaA* line restored the activity of turnercyclamycin B (MIC_90_ 4 µg/mL), reinforcing the role of MlaA in resistance to turnercyclamycin B. We also obtained knockouts of the *E. coli* proteins OmpC and OmpF, which form a complex with MlaA (40, 41). These knockouts were not resistant to turnercyclamycins or colistin, suggesting that the resistance profile observed was specifically due to the interruption of MlaA.

Inspired by these results, we attempted similar experiments with a *pldA* knockout in *Acinetobacter baylyi*, a non-pathogenic strain that is closely related to *A. baumannii*. PldA is the phospholipase that is responsible for degrading mislocalized phospholipids and is independent of the Mla system (43). The Δ*pldA* strain did not significantly demonstrate resistance to turnercyclamycins or colistin and showed similar activity compared to its WT counterpart, *A. baylyi* ADP1 (Fig. S4). Overall, these results revealed that, so far in all mutants tested, only truncation of MlaA was significantly related to resistance to turnercyclamycin B. Further, it was difficult to generate resistance to turnercyclamycin A.

Finally, we aimed to determine whether *mlaA* would similarly affect potency in *A. baumannii*. We obtained three transposon mutants in which the insertion took place at *mlaA* positions 132, 221, and 507 (AB8497, AB8498, and AB8499), respectively. All three insertions were validated both by PCR and by the increase in sensitivity to rifamycin in comparison to the wild type (Fig. 3D). On average, the mutants were potentially slightly more sensitive to colistin in comparison to the wild type. The MIC_90_ of turnercyclamycin B against AB8497 was doubled, while it was increased by fourfold against mutant AB8499. In contrast, turnercyclamycin A retained wild-type potency against these strains. Strain AB8498 had low to no change in MIC_90_s against both turnercyclamycins. These results may indicate that the point of insertion in the *mlaA* gene is important in achieving resistance, as AB8499, which has an insertion at site 507, was the most resistant strain of the three, which is akin to the turnercyclamycin B-resistant DH5α strains, which have an insertion at site 589. The increased resistance when *mlaA* is disrupted in *E. coli* when compared with *A. baumannii* should be addressed in future studies.

### Turnercyclamycin A *in vivo* efficacy

**Fig 4:**
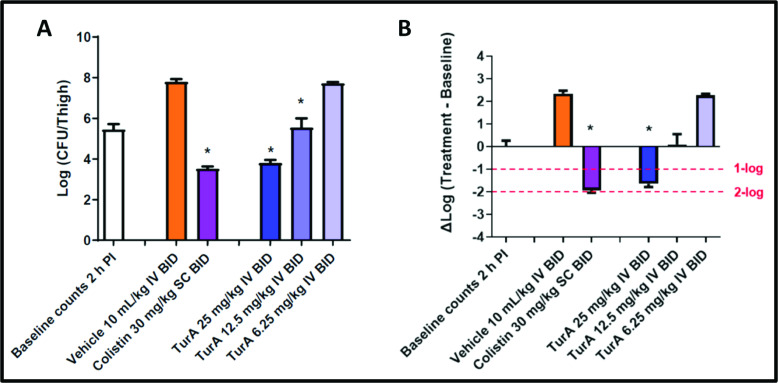
Turnercyclamycin A shows antimicrobial potency *in vivo*. Effects of turnercyclamycin A and colistin in the *A. baumannii* ATCC 17978 thigh infection model with neutropenic BALB/c female mice. (**A**) Bacterial counts from excised thigh tissue for each treatment group. * indicates a significant difference (*P* < 0.05) compared to the respective vehicle control determined by one-way ANOVA followed by Dunnett’s test. (**B**) Change in bacterial counts in thigh tissue at the 26-h sacrifice time point relative to the initial 2-h counts at the time of dosing. * indicates a greater than 1-log10 reduction in counts relative to the baseline at the time of the first dose administration, 2 h after infection, with a significant difference (*P* < 0.05) based on one-way ANOVA followed by Dunnett’s test.

Turnercyclamycin A was produced by fermentation of *T. turnerae* and rigorously purified and quantified. Animal studies were performed at Eurofins. A preliminary pharmacokinetic (PK) and toxicity profile suggested twice-daily dosing at a maximum of 25 mg/kg. The thigh model of infection was employed using *A. baumannii* ATCC 17978, comparing turnercyclamycin A (25, 12.5, and 6.25 mg/kg, i.v. at 2 and 14 h after infection) to colistin (30 mg/kg, s.c. at 2 and 14 h after infection). At 26 h after infection, a dose-dependent response was observed, with a statistically significant reduction in bacterial counts for the higher two doses and a response comparable to colistin for the highest dose (Fig. 4). Further work will elaborate the potential efficacy and any potential liabilities of turnercyclamycins and derivatives.

## DISCUSSION

Complications caused by Gram-negative pathogens are problematic worldwide (1 – 4). These include *Acinetobacter*, which can be difficult to treat, especially in the presence of multidrug resistance (5 – 9). Our initial interest in turnercyclamycins arose because they were specifically effective against Gram-negative pathogens and, more importantly, a panel of resistant *A. baumannii* strains, even those resistant to the last-line agent colistin (30). Here, we show that turnercyclamycin A is effective in an *in vivo* model of *A. baumannii* infection. Because of turnercyclamycin’s clinical potential, it is important to determine why turnercyclamycins had a different resistance profile than colistin. Further, turnercyclamycin A led to less acquired resistance than either colistin or turnercyclamycin B. At a fundamental level, there are several similarities between colistin and turnercyclamycin’s mechanisms of action and resistance, but those were found to lead to cross-resistance under very limited circumstances.

Colistin and turnercyclamycins both required *lpxC* for activity, and that, along with further results, indicated that LPS was crucial to resistance and potentially to the mechanism of action. Multiple types of modifications to LPS are implicated in polymyxin (colistin) resistance. For example, in *Y. pestis*, aminoarabinosylation of one LPS phosphate group leads to resistance to polymyxins (and to turnercyclamycins) (30). In *A. baumannii*, the addition of phosphoethanolamine to a different LPS group by MCR-1 leads to polymyxin resistance but not to turnercyclamycin resistance. We hypothesize that the utility of turnercyclamycins against multidrug-resistant *A. baumannii* may at least in part result from a more prevalent colistin resistance based upon *mcr-1* and not aminoarabinosylation. Further, while loss of LPS itself, as in *lpxC* mutants, could presumably lead to resistance, in *A. baumannii* and many other Gram-negative strains, LPS is important for virulence (44, 45). It remains unclear whether LPS itself is directly involved in the molecular mechanism of action or whether a binding event with LPS is required.

In contrast to polymyxins for which resistant strains are readily selected (46, 47), it was more difficult to obtain resistant mutants to turnercyclamycins, especially to turnercyclamycin A. Turnercyclamycin B-resistant strains shared mutations in *mlaA*, which is involved in the retrograde trafficking of mislocalized phospholipids from the OM to the IM (40, 41). MlaA is not known to directly interact with an antibiotic or to be involved in antibiotic resistance (48 – 51). In a previous study of *A. baumannii* colistin resistance, the co-deletion of LPS/LOS biosynthesis and *mlaA* showed greatly increased resistance in comparison to LPS/LOS loss alone (52). However, the effects of *mlaA* on colistin in *A. baumannii* are subtle and dose dependent. While the deletion of LPS/LOS confers resistance to colistin in *A. baumannii*, the resulting growth defects make *A. baumannii* a less effective pathogen. Recent results show that mutations in other lipid homeostasis genes, especially *mlaA*, are compensatory, enabling regular growth of LPS/LOS mutants (53). Here, we show that turnercyclamycin B itself also selects for *mlaA* mutations in *E. coli* and that turnercyclamycin B loses efficacy in some *mlaA* mutations in *A. baumannii*.

It is striking that turnercyclamycin A was not affected by *mlaA* truncation, when there is only a minor structural difference in the fatty acid tail that distinguishes it from B. This might result either from a direct interaction of MlaA with the lipid portion of the drug or from a differential interaction of turnercyclamycins A and B with phospholipids that are transported by MlaA. In current work in the lipopeptide field, it is common to modify the lipid portion of the molecules and then assume that the underlying mechanisms remain unchanged. Our finding suggests caution, as very simple changes in the turnercyclamycin lipid chains led to a profound change in susceptibility to resistance and to the resistance mechanism itself.

## MATERIALS AND METHODS

### Bacterial strains

The following strains were used: *A. baumannii* ATCC 17978 and ATCC 19606 (American Type Culture Collection, Rockville, MD, USA); DH5α competent cells (Thermo Fisher); *E. coli* C600 (gift of Matthew Mulvey, University of Utah); and *E. coli* BW25113, Δ*ompC,* Δ*ompF*, and Δ*mlaA* (National BioResource Project-*E. coli*, National Institutes of Genetics, Japan). *A. baylyi* ADP1, ADP1 Δ*pldA*, *A. baumannii* 5075 UW, *A. baumannii* Tn26-*mlaA* insertion strains 8497, 8498, and 8499, and *A. baumannii* Δ*lpxC*::ISAbaI were previously generated as described (gift of Colin Manoil, University of Washington) (54). Strains were cryopreserved as single-use cultures at −80°C.

### Antimicrobial broth dilution assay

Cryopreserved strains were streaked on Luria-Bertani (LB) agar (*E. coli*) and Mueller-Hinton (MH) agar (*Acinetobacter*). Plates were incubated at 37°C for 8–12 h. Single colonies were transferred into LB or MH broth and incubated for 6–8 h at 30°C and 150 rpm. Cultures were adjusted to match the 0.5 McFarland standard (1 × 10^8^ cells/mL), diluted 200-fold, and used as the inoculum for assays. Test organisms (200 µL) were added to each well of a 96-well plate, and compounds were added using a twofold dilution scheme starting at 32 µg/mL, with eight dilutions per compound. For Δ*mlaA A. baumannii* strains, 10 µg/mL tetracycline was added, and the compound treatment was a twofold dilution scheme starting at 20 µg/mL, with eight dilutions per compound. After 18–20 h of incubation at 37°C and 150 rpm, 3-(4,5-dimethylthiazol-2-yl)−2,5-diphenyltetrazolium bromide (MTT; 10 µL; 5 mg/mL) was added to each well, followed by incubation for 2 h. MTT formazan was precipitated and then incubated in DMSO (100 µL) for 1 h. *A*_570_ was measured using a Biotek-Synergy 2 Microplate Reader (Biotek).

### LpxC inhibition by CHIR-090 kinetic assay

Single colonies of *A. baumannii* (ATCC 17978) were grown in MH broth and then adjusted to match the 0.5 McFarland standard (1 × 10^8^ cells/mL), which was diluted 200-fold for use as the inoculum. Turnercyclamycins A and B (16 µg/mL) and colistin (8 µg/mL) were used singly and in combination with CHIR-090 (8 µg/mL). Each assay was performed in triplicate in a 96-well plate. The plate was then incubated statically at 37°C in a Biotek-Synergy 2 Microplate Reader (Biotek). The OD_600_ of each well was then measured every 30 min for 24 h.

### Exogenous addition of purified LPS and lipid A assay

*A. baumannii* (ATCC 17978) colonies were grown in MH broth, adjusted to match the 0.5 McFarland standard (1 × 10^8^ cells/mL), and diluted 200-fold for use as the inoculum. Prior to the addition of test compounds, either purified LPS (Sigma-Aldrich L2018) or lipid A (Sigma-Aldrich L5399) (100 µg/mL) was added to wells. Antimicrobial broth dilution assay then proceeded as described previously with turnercyclamycins being treated at a twofold dilution scheme starting at 32 µg/mL, with eight dilutions each, and colistin at a twofold dilution scheme starting at 8 µg/mL, with eight dilutions as a control.

### Antibiotic susceptibility testing of MCR-1-expressing *E. coli*

Chemically competent *E. coli* DH5α and C600 were transformed with empty vector pBR322 (NEB N3033S) and pGDP2-MCR-1 (Addgene 118404), streaked on LB agar under antibiotic selection (kanamycin, 50 µg/mL), and incubated overnight at 37°C. The resulting strains were used in antimicrobial broth dilution assays.

### Evolution of turnercyclamycin-resistant *E. coli* by sublethal dose passaging

Overnight broth cultures of *E. coli* DH5α were diluted 1:500 in LB broth (200 µL) containing 0.5×, 1×, and 2× MIC of each turnercyclamycin and incubated at 30°C and 150 rpm for 24 h. The highest concentration that showed growth based upon turbidity was adjusted to the 0.5 McFarland suspension and diluted 200-fold to be used as the inoculum for the next passage. The concentration of the drug that was twofold above the previous inoculum was assigned as the new 1× MIC. This was repeated until an MIC of 32 µg/mL was achieved. Resistance was confirmed and maintained by growing it in LB containing the compound with the new MIC. Experiments were performed in four independent cultures. The purity of the resistant strains was confirmed by wet mount microscopy, 16S rDNA sequencing, and metagenomics analysis via Autometa v2.2.0 (55). Upon purity analysis, one strain was found to be contaminated with *Staphylococcus* (Strain 2), as determined by Autometa binning.

### Next-generation sequencing and mutation analysis

Illumina library preparation and sequencing were performed at the Huntsman Cancer Institute-High-Throughput Genomics (HCI-HTG) shared resource at the University of Utah. Sequencing library preparation used an NEBNext Ultra II DNA Library Prep Kit with a 450-bp mean insert size. Sequencing used an Illumina NovaSeq 6000 sequencer with 2× 150-bp runs. Raw reads were trimmed by Trimmomatic and assembled with metaSPAdes (56). Genes were predicted using Prodigal (57), and coding sequences were compared between wild-type and mutant strains using NCBI blastn tools. The threshold for identifying the same gene was set at 90% identity in the nucleotide sequence. Any gene in the mutant strain genome that had an identity of less than 100% or an alignment coverage of less than 100% when compared to the gene from the wild-type strain was considered a mutated gene. Gene alignment used CLC Genomics Workbench 11 and Geneious v10.2.2.

### Plasmid complementation for Δ*mlaA E. coli*

*E. coli* BW25113 and Δ*mlaA* were transformed with a medium copy number pTwist Amp plasmid expressing MlaA (Twist Bioscience, pTwistAmp_MlaA; Fig. S6) and used in antimicrobial broth dilution assays described above, except that kanamycin (0.1 mg/mL) and ampicillin (0.1 mg/mL) were added to the mixtures.

### Quantification and statistical analysis

MIC data and growth curves were graphed using GraphPad Prism 9.5.1. Error bars on MIC graphs and growth curves were indicated as mean ± SD of three replicate wells and are done in either two or three biological replicates.

### Animal studies

Eurofins used mice from BioLASCO Taiwan. ALB/c female mice (18 ± 2 g) and BALB/c female mice (7 wk old) were used for PK/toxicity and efficacy studies, respectively. The study was conducted at Eurofins (Taiwan), an AAALAC-accredited facility. The protocol was reviewed by the IACUC at Pharmacology Discovery Services Taiwan, Ltd. The University of Utah IACUC number associated with this study is 22-01003.

### Pharmacokinetics

Protocols were performed at Eurofins (Taiwan). Turnercyclamycin A in plasma was measured by liquid chromatography-tandem mass spectrometry, and graphs illustrating the mean plasma concentrations (mean ± SD) over time were generated. PK parameters were obtained using non-compartmental analysis of the plasma data with WinNonlin.

### Neutropenic thigh infection model

Five neutropenic mice were used in each treatment group. The mice were anesthetized with 3%–5% isoflurane inhalation and then injected intramuscularly in the left thigh with a 0.1-mL suspension of *A. baumannii* ATCC 17978 [1.9 × 10^5^ colony-forming units (CFU) per mouse]. At 2 and 14 h after infection, turnercyclamycin A was administered intravenously slowly (~30 s, IV bolus) at doses of 6.25, 12.5, and 25 mg/kg. Subcutaneous administration of colistin at a dose of 30 mg/kg was also performed at the same time points. The dosing volumes were 10 mL/kg. An infected control group, which did not receive any treatment, was sacrificed at 2 h after infection to establish the baseline bacterial counts. The remaining groups were sacrificed at 26 h. Bacterial counts in the thigh, standardized to the weight of each thigh, were determined using a serial dilution plating method and calculated using the formula below:


$$Decrease\;\left(\%\right)=\;\frac{\left(CFU\;per\;thigh\;of\;vehicle-CFU\;per\;thigh\;of\;treatment\right)}{CFU\;per\;thigh\;of\;vehicle}\;\times \;100\%$$

Statistical significance (*P* < 0.05) was evaluated using one-way ANOVA followed by Dunnett’s test through GraphPad Prism software version 5.0. A significant decrease in bacterial counts, with a *P* value < 0.05, compared to the vehicle control or baseline groups, was considered statistically significant.

## SUPPLEMENTAL MATERIAL

Supplemental Material spectrum.02306-23-s0001.pdf Supplemental Figures and Tables.

ASM does not own the copyrights to Supplemental Material that may be linked to, or accessed through, an article. The authors have granted ASM a non-exclusive, world-wide license to publish the Supplemental Material files. Please contact the corresponding author directly for reuse.
