# Supplementary material for: Resistance mechanisms for Gram-negative bacteria-specific lipopeptides, turnercyclamycins, differ from that of colistin
Source: Microbiol Spectr. 2023 Oct 26;11(6):e02306-23. doi: 10.1128/spectrum.02306-23 (PMC10714751; doi:10.1128/spectrum.02306-23)
Supplement: Supplemental Material — Supplemental Figures and Tables. [file spectrum.02306-23-s0001.pdf]

SUPPLEMENTAL INFORMATION for:

Resistance mechanisms for Gram-negative bacteria-specific lipopeptide, turnercyclamycins, differ from that of colistin

Albebson L. Lim[a], Bailey W. Miller[a], Zhenjian Lin[a], Mark A. Fisher[b], Louis R. Barrows[c], Margo G. Haygood[a], Eric W. Schmidt[a#]

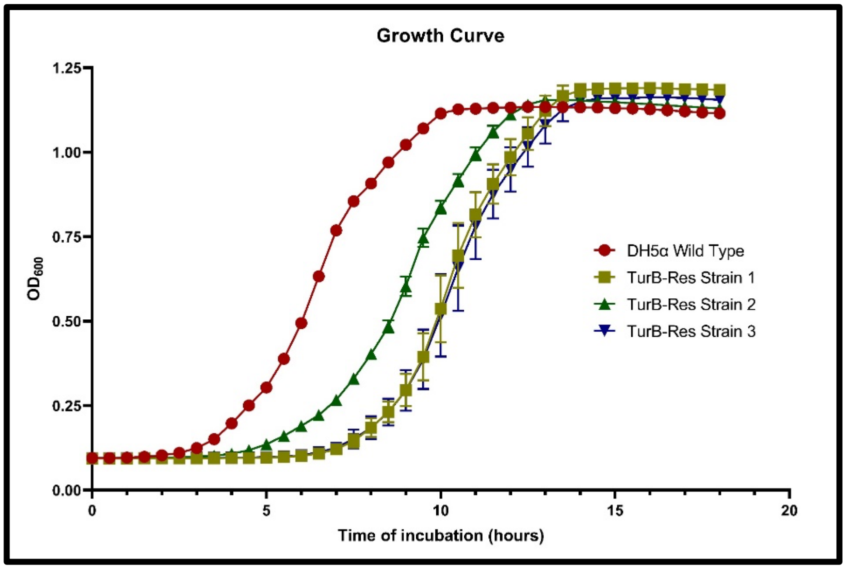

Figure S1. Growth curve of wild type and turnercyclamycin B-resistant *E. coli* DH5α strains.

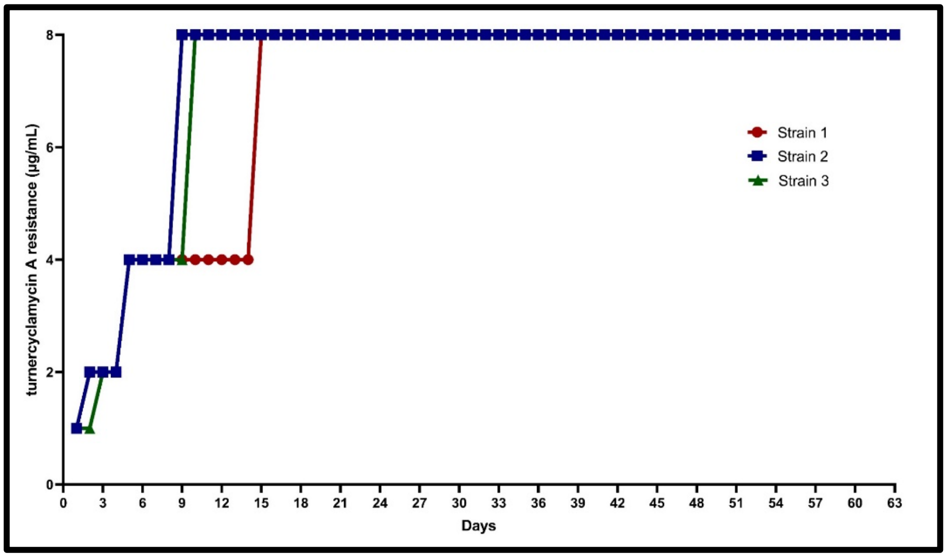

Figure S2. Turnercyclamycin A-resistant mutants were generated by daily serial passaging of *E. coli* DH5α at sub-MIC concentrations of turnercyclamycin, leading to a steady shift in MIC of turnercyclamycin A. This experiment was performed in three biologically independent samples.

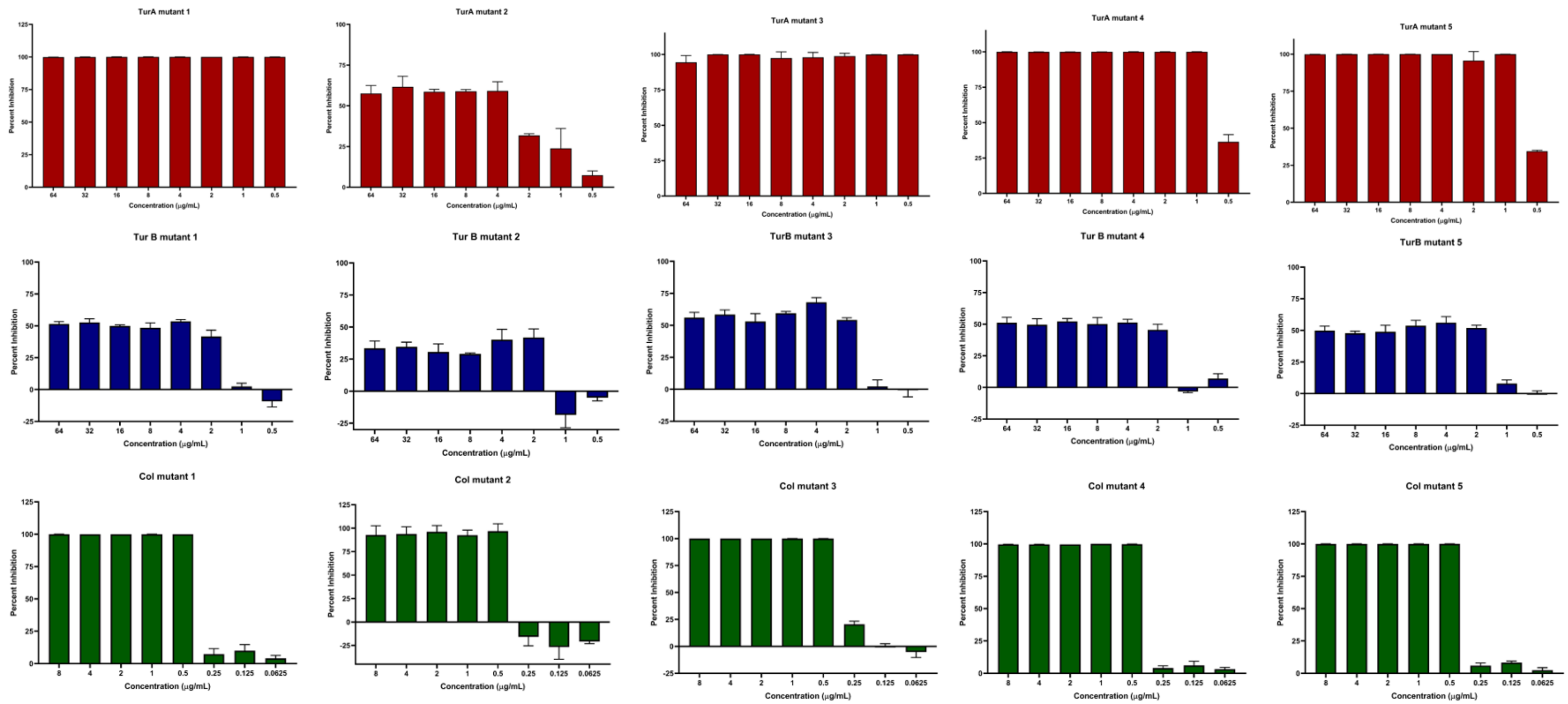

Figure S3. MICs against turnercyclamycin B-resistant *E. coli* DH5α using turnercyclamycin A, turnercyclamycin B, and colistin.

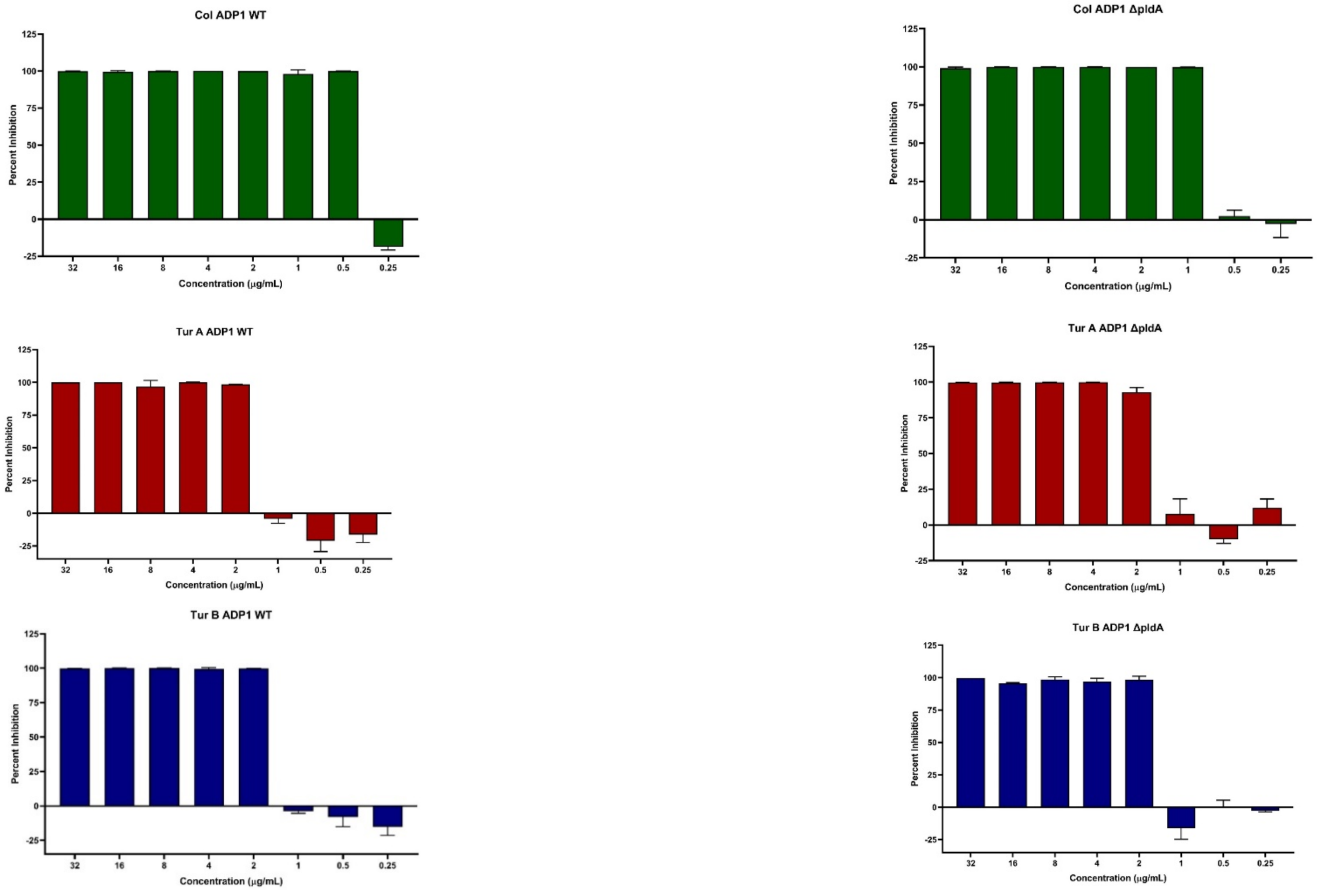

Figure S4. MICs of turnercyclamcyins A and B, and colistin against WT ADP1, and Δ*pldA Acinetobacter baylyi* strains.

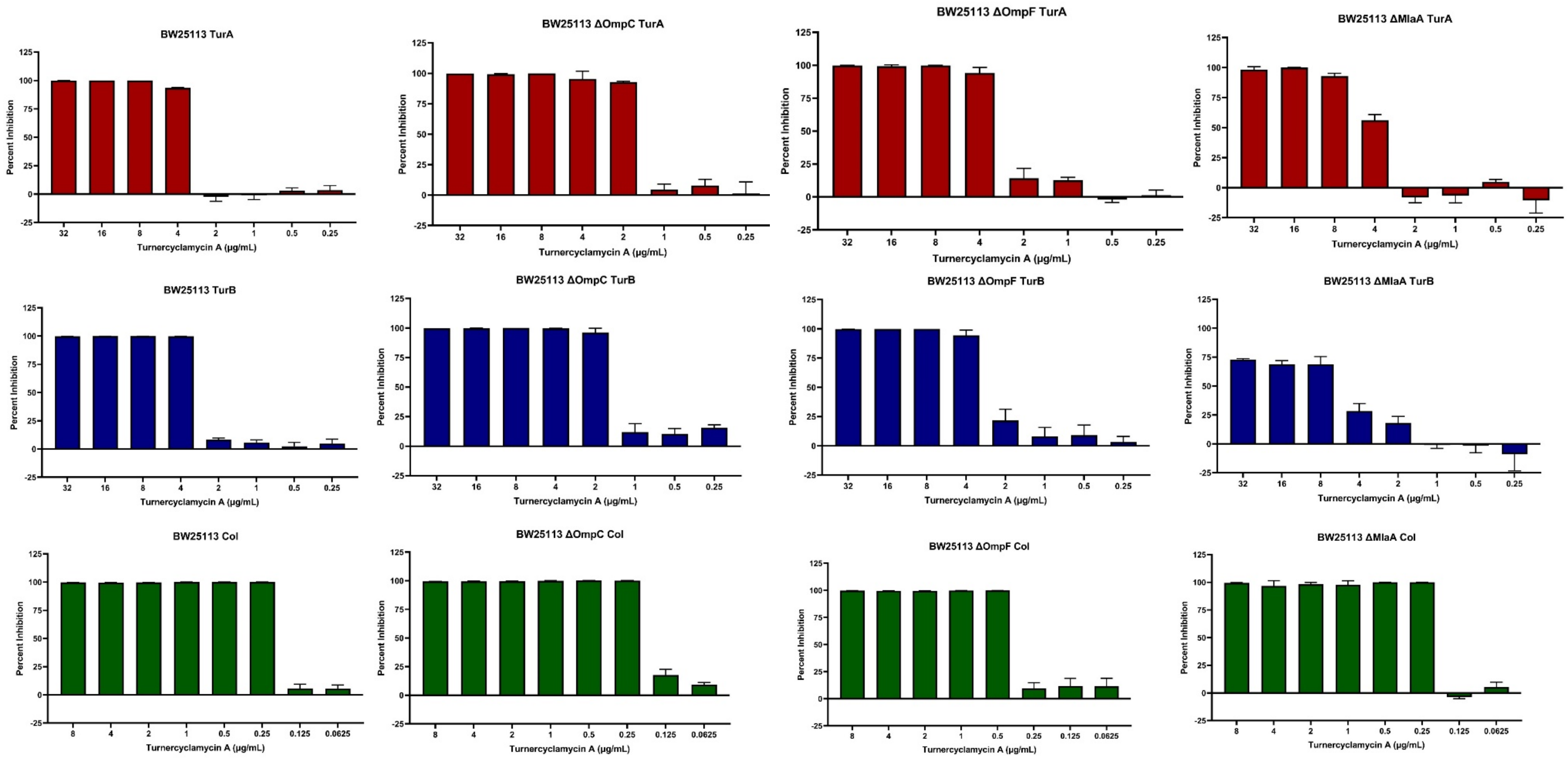

Figure S5. MICs of Keio strains BW25113 WT, Δ*ompC*, Δ*ompF*, and Δ*mlaA E. coli* strains against turnercyclamcyins A and B, and colistin.

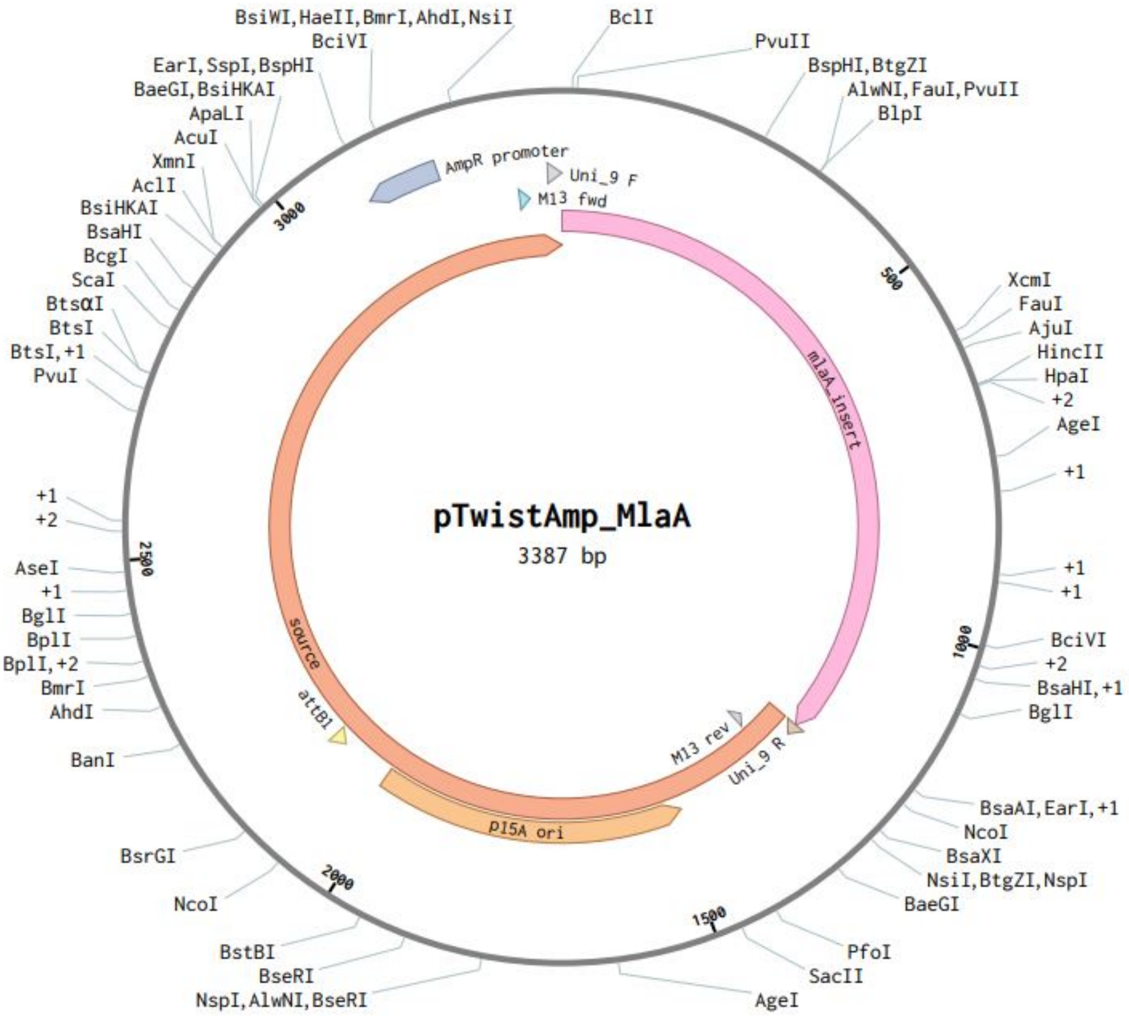

Figure S6. Plasmid map of pTwistAmp_MlaA, complementation plasmid expressing MlaA protein.

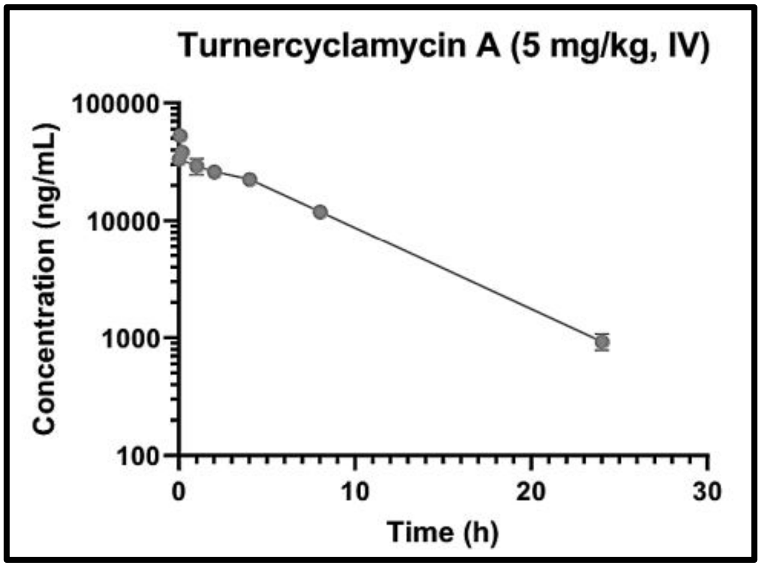

Figure S7. Plasma concentration collected during the duration of the PK experiments.

Table S1: Final results per mouse of thigh infection efficacy study. *indicates significant difference between experimental counts and baseline counts. #indicates significant difference between experimental counts and vehicle control.

Group	Treatment	Dose Route / Schedule	Animal No.	Thigh Weight (g)	CFU/thigh Time post inoculation 2, 26 h	Decrease (%)	Log (CFU/thigh) Time post inoculation 2, 26 h	Δ
1	Baseline, 2 h post infection	N/A	1	0.741	4.20×10^5		5.62	
			2	0.683	6.99×10^5		5.84	
			3	0.766	4.14×10^5		5.62	
			4	0.798	5.91×10^5		5.77	
			5	0.748	2.64×10^4		4.42	
			Mean	0.747	4.30×10^5	--	5.45	--
			SEM	0.019	1.14×10^5		0.26	
2	Vehicle (3% ethanol/ PBS)	10 mL/kg IV, BID, q12h	1	0.725	1.21×10^8		8.08	
			2	0.738	2.96×10^7		7.47	
			3	0.826	4.10×10^7		7.61	
			4	0.813	1.30×10^8		8.11	
			5	0.751	5.40×10^7		7.73	
			Mean	0.771	7.51×10^7	--	7.80	2.35
			SEM	0.020	2.10×10^7		0.13	
3	Colistin	30 mg/kg SC, BID, q12h	1	0.718	1.77×10^3		3.25	
			2	0.652	5.73×10^3		3.76	
			3	0.648	5.64×10^3		3.75	
			4	0.644	1.98×10^3		3.30	
			5	0.739	3.57×10^3		3.55	
			Mean	0.680	3.74×10^3	100	3.52*	-2.73#
			SEM	0.020	8.54×10^2		0.11	
4			1	0.644	5.91×10^3		3.77	

Group	Treatment	Dose Route / Schedule	Animal No.	Thigh Weight (g)	CFU/thigh Time post inoculation 2, 26 h	Decrease (%)	Log (CFU/thigh) Time post inoculation 2, 26 h	Δ
	PT# 1251595 (UUM-2) (Turnercyclamycin A)	25 mg/kg IV, BID, q12h	2	0.691	7.68×10^3		3.89	
			3	0.602	8.76×10^3		3.94	
			4	0.713	1.38×10^4		4.14	
			5	0.709	1.89×10^3		3.28	
			Mean	0.672	7.61×10^3	100	3.80*	-1.65[#]
			SEM	0.021	1.94×10^3		0.14	
5	PT# 1251595 (UUM-2) (Turnercyclamycin A)	12.5 mg/kg IV, BID, q12h	1	0.783	5.67×10^5		5.75	
			2	0.803	7.02×10^6		6.85	
			3	0.771	7.89×10^5		5.90	
			4	0.766	1.32×10^5		5.12	
			5	0.752	1.35×10^4		4.13	
			Mean	0.775	1.70×10^6	98	5.55*	0.10
			SEM	0.009	1.34×10^6		0.45	
6	PT# 1251595 (UUM-2) (Turnercyclamycin A)	6.25 mg/kg IV, BID, q12h	1	0.782	4.59×10^7		7.66	
			2	0.821	3.63×10^7		7.56	
			3	0.788	4.62×10^7		7.66	
			4	0.753	6.93×10^7		7.84	
			5	0.000	7.71×10^7		7.89	
			Mean	0.629	5.50×10^7	27	7.72	2.27
			SEM	0.158	7.76×10^6		0.06	

Table S2. PK parameters of turnercyclamycin A after 5mg/kg IV administration. $t_{1/2}$: halftime; C: concentration; AUC: area under the curve; D: dose; MRT: mean residence time; VSS: Vd at steady state; CL: clearance rate.

$t_{1/2}$ (h)	C0 (ng/mL)	AUClast (h*ng/mL)	AUCInf (h*ng/mL)	AUC/D (h*kg*ng/mL/mg)	AUC Extr (%)	MRT (h)	VSS (L/kg)	CL (ml/h/kg)
4.35	33657	284562	290402	58080	2.01	5.83	0.10	17.4
